# EUS-guided drainage using lumen apposing metal stent and percutaneous endoscopic necrosectomy as dual approach for the management of complex walled-off necrosis: a case report and a review of the literature

**DOI:** 10.1186/s13017-021-00367-y

**Published:** 2021-06-02

**Authors:** Cecilia Binda, Monica Sbrancia, Marina La Marca, Dora Colussi, Antonio Vizzuso, Matteo Tomasoni, Vanni Agnoletti, Emanuela Giampalma, Luca Ansaloni, Carlo Fabbri

**Affiliations:** 1 Gastroenterology and Digestive Endoscopy Unit, Forlì-Cesena Hospitals, AUSL Romagna, Forlì-Cesena, Italy; 2 Gastroenterology Unit, Department of Medical and Surgical Sciences, S.Orsola-Malpighi Hospital, Bologna, Italy; 3 Radiology Unit, Morgagni-Pierantoni Hospital, AUSL Romagna, Forli, Italy; 4 General, Emergency and Trauma Surgery Department, M. Bufalini Hospital, Cesena, Italy; 5 Anesthesia and Intensive Care Unit, M. Bufalini Hospital, AUSL Romagna, Cesena, Italy; 6 Radiology Unit, M. Bufalini Hospital, AUSL Romagna, Cesena, Italy

**Keywords:** Necrotizing pancreatitis, Percutaneous endoscopic necrosectomy, Walled-off pancreatic necrosis, Endoscopic necrosectomy, Lumen-apposing metal stent

## Abstract

**Background:**

Endoscopic ultrasound-guided drainage is suggested as the first approach in the management of symptomatic and complex walled-off pancreatic necrosis. Dual approach with percutaneous drainage could be the best choice when the necrosis is deep extended till the pelvic paracolic gutter; however, the available catheter could not be large enough to drain solid necrosis neither to perform necrosectomy, entailing a higher need for surgery. Therefore, percutaneous endoscopic necrosectomy through a large bore percutaneous self-expandable metal stent has been proposed.

**Case presentation:**

In this study, we present the case of a 61-year-old man admitted to our hospital with a history of sepsis and persistent multiorgan failure secondary to walled-off pancreatic necrosis due to acute necrotizing pancreatitis. Firstly, the patient underwent transgastric endoscopic ultrasound-guided drainage using a lumen-apposing metal stent and three sessions of direct endoscopic necrosectomy. Because of recurrence of multiorgan failure and the presence of the necrosis deeper to the pelvic paracolic gutter at computed tomography scan, we decided to perform percutaneous endoscopic necrosectomy using an esophageal self-expandable metal stent. After four sessions of necrosectomy, the collection was resolved without complications. Therefore, we perform a revision of the literature, in order to provide the state-of-art on this technique. The available data are, to date, derived by case reports and case series, which showed high rates both of technical and clinical success. However, a not negligible rate of adverse events has been reported, mainly represented by fistulas and abdominal pain.

**Conclusion:**

Dual approach, using lumen apposing metal stent and percutaneous self-expandable metal stent, is a compelling option of treatment for patients affected by symptomatic, complex walled-off pancreatic necrosis, allowing to directly remove large amounts of necrosis avoiding surgery. Percutaneous endoscopic necrosectomy seems a promising technique that could be part of the step-up-approach, before emergency surgery. However, to date, it should be reserved in referral centers, where a multidisciplinary team is disposable.

## Background

Acute pancreatitis could be complicated by necrosis of the pancreatic gland or peripancreatic tissue in 10–20% of cases [1, 2]. The subset of patients that develop necrosis and superadded infection of the necrotic tissue has a mortality rate that could rank 30% if they are untreated and 6.7% if drainage is performed [1]. Therefore, interventional procedures, especially endoscopic ultrasound (EUS)-guided drainage, are to date recommended by guidelines for the treatment of symptomatic pancreatic fluid collections (PFCs) [2, 3].

In the last decade, lumen apposing metal stents (LAMS) have been put into the market, favoring the development of EUS-guided drainage and facilitating direct endoscopic necrosectomy (DEN). When the extension of the necrosis to the pelvic paracolic gutter is present, a transmural endoscopic drainage could be insufficient and a dual approach using percutaneous drainage is recommended [2, 3]. However, even when large catheters are used, the percutaneous treatment could represent an unsatisfactory gateway for solid necrosis. On the other hand, surgical necrosectomy, even though a minimally invasive approach, is burdened by high mortality and complication rates [4]. Therefore, a percutaneous endoscopic necrosectomy (PEN) through self-expandable metal stent (SEMS) has been proposed, showing promising results [5–8].

In this study, we proposed a dual approach with EUS-guided drainage using LAMS and percutaneous drainage using a large bore SEMS for the treatment of a symptomatic walled-off pancreatic necrosis (WOPN) extended to the pelvic paracolic gutter. Moreover, a comprehensive review of the literature has been performed, in order to provide an overview of the available evidences of this technique.

## Case presentation

**Fig. 1:**
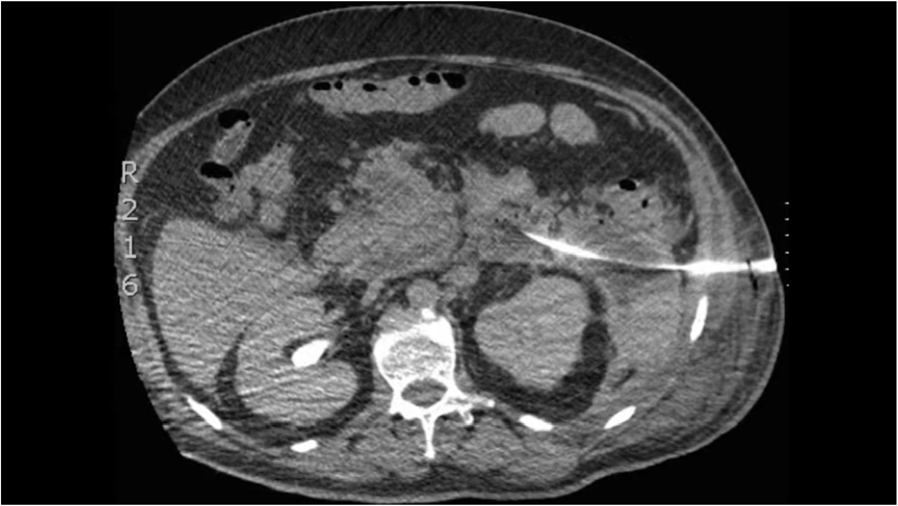
CT scan previous percutaneous SEMS placement

**Fig. 2:**
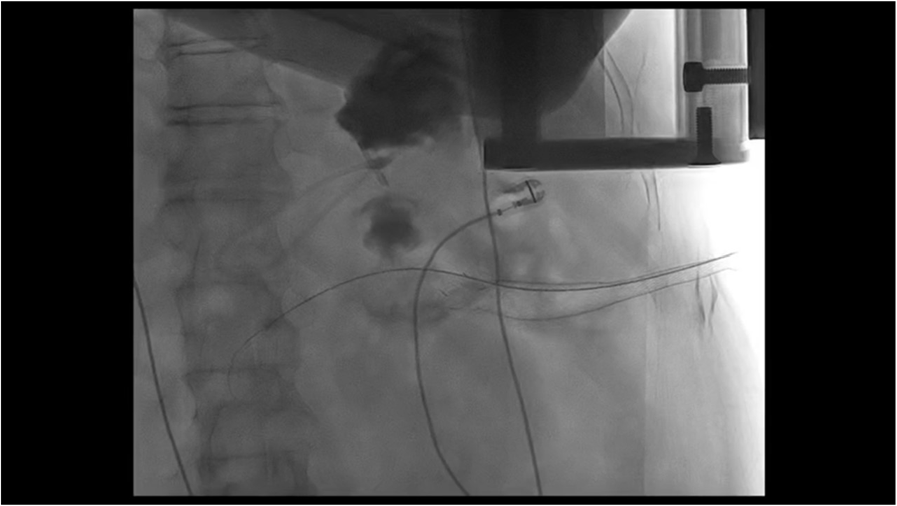
Radiological view of the EC-LAMS and of the percutaneous stent placed on the left side of the abdomen

**Fig. 3:**
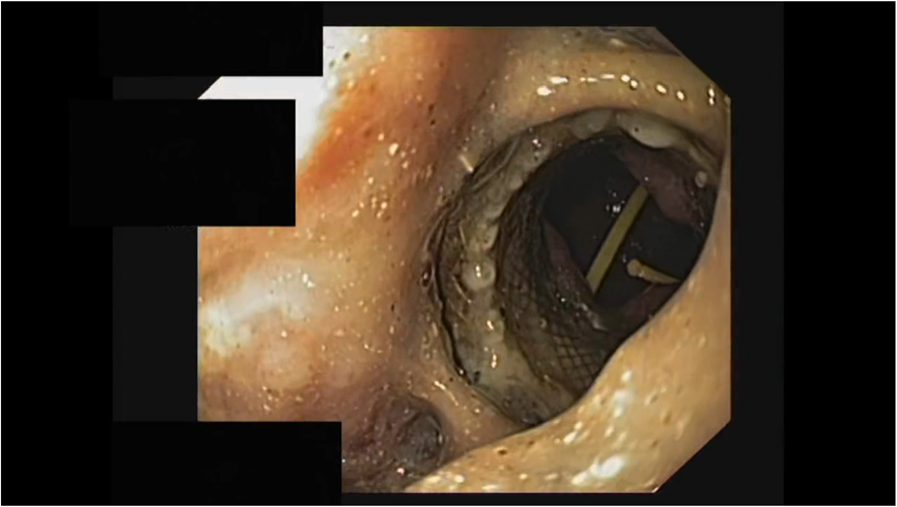
Endoscopic appearance of the cavity after SEMS placement, with view of the EC-LAMS in place

A 61-year-old man developed signs of severe sepsis with multiorgan failure (MOF) 3 weeks after the onset of an acute necrotizing pancreatitis (ANP). A computed tomography (CT) scan revealed the presence of a large WOPN with signs of infection and the patient underwent EUS-guided drainage using LAMS 20 × 10mm (Hot-Axios, Boston Scientific Corp., Marlborough, MA, USA). Three sessions of DEN were performed and the patient rapidly recovered, although a large amount of necrosis was still remnant. After 3 weeks from LAMS placement, the patient newly developed MOF and septic shock; a new CT scan showed an increased amount of necrosis extended deep to the left pelvic paracolic gutter. Because of the severe clinical conditions, surgery was excluded and we decided to perform a percutaneous drainage using a large bore SEMS. Using CT guidance, a catheter was inserted on the left side of the abdomen reaching the necrotic collection (Fig. 1); therefore, the tract was balloon dilated and an esophageal SEMS (TaeWoong Niti-S 20 × 100 mm) was placed over a guidewire (Fig. 2). A balloon-dilation on the stent was done in order to allow the entrance of the gastroscope within the collection (Fig. 3), and a first session of PEN was performed using a snare, leading a rapid resolution of the sepsis. Four sessions of PEN were completed; between each session, irrigation with saline solution and instillation of antibiotics and amphotericin were performed.

**Fig. 4:**
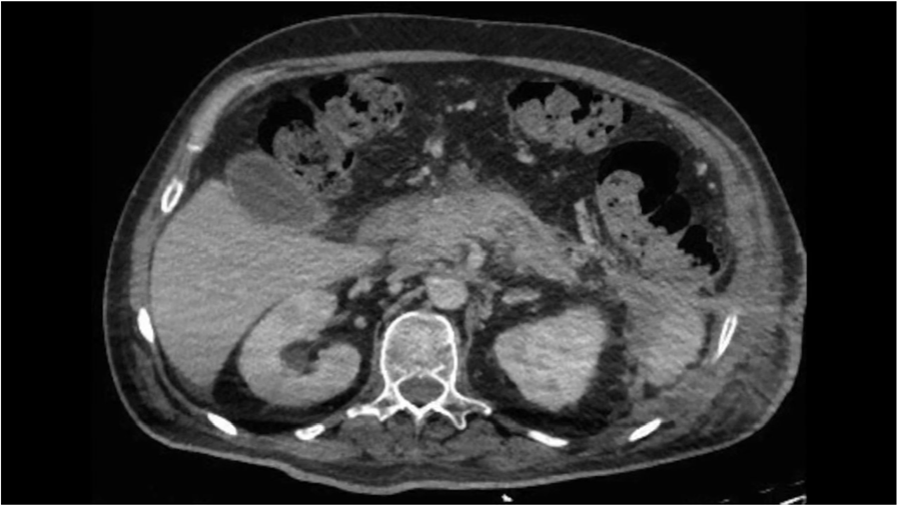
CT scan after SEMS removal showing a complete resolution of the collection

Two weeks after SEMS placement, the LAMS was pulled out, while the percutaneous stent was removed 1 week later, when a complete resolution of the necrosis was obtained (Fig. 4). The large cutaneous bore fistula was sutured and medicated for two months. The patient was discharged 3 weeks later after SEMS removal. No immediate or late complications occurred. At long-term follow-up, 557 days, the patient is asymptomatic, without evidence of recurrence of the collection.

## From sinus tract endoscopy to SEMS-assisted percutaneous necrosectomy for the treatment of WOPN: a literature overview

In the early 2000s, Carter and colleagues described the development of a minimally invasive approach to retroperitoneal/peripancreatic necrosis, using percutaneous endoscopic necrosectomy (PEN, often referred to as *sinus tract endoscopy*) for the debridement of solid necrotic tissue with either a flexible or a rigid endoscopic system [9]. Ten patients were managed using a percutaneous approach plus PEN as the primary treatment. Except for two patients who died from MOF, the remaining eight patients recovered without the need of adjuvant open surgical treatment, providing an 80% success rate. Moreover, 60% of patients were treated outside the intensive care unit (ICU), because they did not require organ support.

Technically, percutaneous endoscopic necrosectomy was preceded by the placement of a percutaneous drainage catheter by interventional radiologists, typically providing an immediate relief of symptoms by decompressing the fluid part of the collection, but that can be inadequate in case of consistent solid necrotic tissue, thus resulting in persistence of sepsis. After the stabilization of the percutaneous tract, a sequential use of upsizing drain (over 28Fr) or dilatation using a CRE™ balloon was performed, allowing the introduction of a pediatric or adult standard upper endoscope into the collection. The necrotic cavity was initially inspected through serial lavages with sterile normal saline and CO_2_ insufflation; then, several endoscopic devices can be used to remove the necrotic tissue, such as rat-tooth forceps, polypectomy snare, and Dormia basket.

After this first experience, several studies and case reports regarding percutaneous endoscopic necrosectomy have been published [10–17], identifying in the above technique an efficient and safe alternative to video-assisted retroperitoneal debridement (VARD) in the treatment of infected WOPN located distal from the gastrointestinal tract. In their retrospective analysis, Moyer et al. [16] reported a clinical success rate of about 82% (19/23), represented by 1-year sustained resolution of symptoms and fluid collection. Likewise, Jain and colleagues [17], whom recently published the largest observational cohort study including 53 patients with either acute, infected necrotic collections or WOPN, achieved a 77% clinical success rate. Indeed, about 12 out of 53 patients (23%) required additional surgical necrosectomy due to persistence of sepsis and organ failure. Post-procedural adverse events varied widely across the studies, reaching a 25% rate at follow-up [16], which was similar to those recently reported in a meta-analysis of three randomized control trials (RCTs) comparing the clinical outcomes between endoscopy (using EUS-guided drainage via cystogastrostomy or cystoenterostomy) and minimally invasive surgery treatment for necrotizing pancreatitis [18].

As we have already highlighted, the abovementioned technique is not free from complications. In fact, in order to gain a wide opening access to the collection, repeated balloon dilatations are required, carrying not only the risk of bleeding [5], but also to postpone the necrosectomy session until the maturity of the skin tract. Together with the need of multiple PEN sessions to achieve the complete debridement of necrotic tissue, these limitations flowed into the use of covered esophageal SEMS as firstly described in 2011 by Navarrete et al. [5], allowing to easily achieve and maintain a stable access to the cavity. Several other case reports and studies [6–8, 19–26] describing PEN through SEMS for the management of complex WOPN were published thereafter, summarized in Table 1.

**Table 1:** Overview of studies and case reports using SEMS for percutaneous endoscopic necrosectomy

Study, year of publication	Patients,n	Mean age, y	Type of collection	Size of collection, mean, cm (range)	Previous endoscopic/sur-gical treatment attempt (n)	Mean number of PEN sessions performed	Length of time stent in place, mean, days	Peri-procedural complications, type (n)	Additional surgical intervention for incomplete debridement,n	Clinical success and outcomes
Pérez Cuadrado Robles, 2020	1	72	WOPN	23	LAMS placement, percutaneous catheter drainage	2	N/A	None (0)	0	100% clinical success
Ke, 2019	23	43	WOPN	N/A	Percutaneous catheter drainage (23)	2	7	Minor bleeding conservatively treated (1)	5	11 patients (48%) had either a major complication and/or died (e.g. septic shock and MOF, hemorrhagic shock), pancreatic fistula (2)
Tringali, 2018	3	45	WOPN	15 (7-20)	Percutaneous catheter drainage (3)	3	12.7	None (0)	0	Fluid collection’s recurrence with fever (1)
Thorsen, 2018	5	44	WOPN	33.4 (26-54)	Percutaneous catheter drainage (3), ETDN (2)	5.75	37.5	None (0)	0	Septic shock and death (1), productive cutaneous fistula (2)
Saumoy, 2017	9	62	WOPN	11.2 (4.7-17.1)	Percutaneous catheter drainage (9)	3	14.7	None (0)	0	MOF and death (1)
D’Souza, 2017	1	32	WOPN	N/A	Percutaneous catheter drainage	1	N/A	None (0)	0	100% clinical success
Sato, 2016	1	13	WOPN	N/A	Percutaneous catheter drainage	3	17	None (0)	0	100% clinical success
Kedia, 2015	1	56	WOPN	17	Percutaneous catheter drainage, ETDN	2	N/A	None (0)	0	100% clinical success
Ceredo-Rodriguez, 2014	1	43	WOPN	N/A	Percutaneous catheter drainage, surgical lavages	7	N/A	None (0)	0	100% clinical success
Bakken, 2011	2	70	WOPN	N/A	N/A	2.5	25	1	N/A	N/A
Bakken, 2011	1	75	WOPN	N/A	Percutaneous catheter drainage	N/A	N/A	None (0)	N/A	N/A
Navarrete, 2011	1	37	Infected pseudocyst	N/A	Percutaneous catheter drainage	4	12	None (0)	0	100% clinical success

*PEN* percutaneous endoscopic necrosectomy, *WOPN* walled-off pancreatic necrosis, *MOF* multi-organ failure, *PD leak* pancreatic duct leak, *ETDN* endoscopic trans-gastric drainage and necrosectomy

**Fig. 5:**
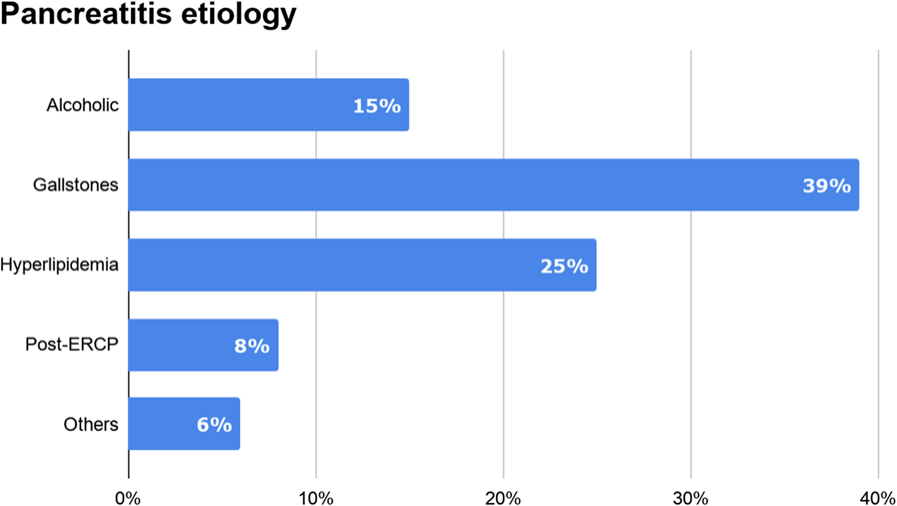
Distribution of pancreatitis etiology in SEMS-assisted percutaneous endoscopic necrosectomy studies

Considering all the published studies, 49 patients have been treated using this technique, with a technical success of 100% [5–8, 19–26]. Regarding the etiology of ANP, summarized in Fig. 5, gallstones emerged as the most common cause, accounting for almost 40% of cases (19/48), which is in line with data derived from Western countries [27].

Although some differences regarding the definition of clinical success has emerged, the referred rates ranged from 65 to 89% [6, 8, 25]. The number of PEN sessions needed for WOPN resolution varied widely, from 1 to 7, and the mean time for stent removal ranged between 7 and 37 days (range 3–65) [5–8, 19–26]. Additional minimally invasive debridement procedures (i.e., percutaneous and/or trans-gastric, even simultaneous) was required in up to 65% of patients [8, 25], while 30% of patients (7/23) received additional open surgery as reported by Ke and colleagues [25], most of them (5/23) because of inadequate endoscopic debridement of necrotic tissue.

Furthermore, most of the studies reported a wide range of several complications occurred during the hospitalization, including enteric/colonic fistula, compartment syndrome with muscle necrosis, and ongoing septic shock requiring invasive life support measures, mainly related to the clinically severe form of ANP. Indeed, no severe, procedure-related adverse events were observed, and the rate of pancreatic fistula was significantly lower compared to the surgical approach, as emerged in the TENSION trial [4–8, 19–26]. However, this translates into a lengthening of hospitalization as emerged in Thorsen et al. study [6]. Patients included in this series were discharged after a mean of 61 days after percutaneous SEMS removal, although the length of the hospital stay varied among patients and were strongly influenced by the several complications occurred (i.e., stomach and colon perforation after ETDN attempt, aspiration pneumonia, critical illness neuropathy etc.). In the case series by Tringali and colleagues [7], the median length of hospitalization after esophageal stent placement was 18 days, similar with those reported by Navarrete and Cerecedo-Rodriguez and colleagues (15 and 21 days, respectively) [5, 21].

## Discussion

Complex WOPN and extended necrotic tissue without a mature capsule are hard-to-treat conditions and potentially life-threatening. Nevertheless, a standard technique is not yet defined, and it is quite clear that a single approach could be unsatisfactory. In fact, it has become increasingly evident that a multidisciplinary, step-up strategy in the treatment of symptomatic fluid collections and infected WOPN leads to better clinical outcomes and lower rates of adverse events than more aggressive treatments [4, 28, 29]. As stated by various international guidelines [2, 3], percutaneous drainage (PCD) or endoscopic transluminal drainage actually represent the initial step, based on location of the necrotic collections and local expertise, and a dual approach is suggested, especially when the necrosis is extended deep to the pelvic paracolic gutter. PCD has the advantage of being widely available and can provide immediate relief of symptoms in those patients who are too ill to undergo endoscopic maneuver, often acting as the first drainage procedure of choice, particularly when necrosis is located distal from both stomach and duodenum. However, solid necrotic tissue cannot be effectively evacuated by small caliber percutaneous catheters and frequently requires direct debridement for complete resolution. To date, therefore, several endoscopic options of treatment of necrotic collections have been proposed and are outlined in Table 2.

**Table 2:** Endoscopic treatment options for the management of pancreatic fluid collections

Procedure	Gateway	Type of stent and diameter	Advantages	Limitations
Transmural endoscopic drainage with plastic stents	• Cystogastrostomy • Cystoduodenostomy	Double-pigtail 7-10 Fr	• Low cost • Easy placement and removal • Active or passive drainage (with or without nasocystic drain)	• Small caliber (increased risk of occlusion and secondary infection) • Often need for multiple stents • Possibility of fluid leak and migration • Poor visibility under fluoroscopy during the procedure
Transmural endoscopic drainage with FCSEMSs	• Cystogastrostomy • Cystoduodenostomy	Tubular biliary stents 6-10 mm	• Large caliber • Possibility to perform DEN through stent • Prevent fluid leak • Good visibility under fluoroscopy • Haemostatic effect at puncture site	• Cost • Difficult placement • Increased risk of stent migration and delayed bleeding (even for the possibility of GI tract injury)
Transmural endoscopic drainage with LAMSs	• Cystogastrostomy • Cystoduodenostomy	• AXIOS™, 10-15-20 mm (Boston Scientific, Marlborough, MA, USA) • SPAXUS™, 8-10-16 mm (Taewoong Medical, Gimpo, Korea) • NAGI™, 10-16 mm (Taewoong Medical, Gimpo, Korea) • AixstentⓇ PPS, 10-15 mm (Leufen Medical, Berlin, Germany) • Hanaro stentⓇ, 10 mm (Mi-TECH-Medical Co, Seoul, South Korea)	• Large caliber • Easy placement, even without the need for wire exchange • Easy removal • Possibility to perform DEN through stent • Lower risk of migration • Reduced need for fluoroscopy and nasocystic drain placement	• Cost • Increased risk of suprainfection and bleeding in the early phases (<14 days)
Transpapillary drainage via ERCP	• Major/minor duodenal papilla	Pancreatic endoprosthesis 5-7 Fr	• Active or passive drainage (with or without nasal drain) • Evidence of pancreatic duct disruption • Considered as an alternative when the distance from the GI wall to the PFC is too large (>10-15 mm)	• Limited use in WOPN with poorly-liquefied necrotic tissue
Percutaneous endoscopic necrosectomy	• Percutaneous approach	Esophageal, partially or fully-covered SEMS 18-20 mm	• Single or multiport gateway • Possibility to perform DEN even if PFC is located distal from the GI tract • Reduced need of deep sedation	• Preceded by the creation of cutaneous fistula by interventional radiologists • Increased risk of peri-procedural bleeding

*FCSEMS* fully-covered self-expandable metal stents, *DEN* direct endoscopic necrosectomy, *GI* gastro-intestinal, *LAMSs* lumen-apposing metal stents, *ERCP* Endoscopic retrograde cholangiopancreatography, *PFC* pancreatic fluid collection

We believe that placement of a large bore percutaneous SEMS allows to overcome these limitations by performing direct necrosectomy with either a standard or therapeutic flexible endoscope, which offers greater maneuverability and penetration into deep recesses than VARD. This technique might also lead to faster debridement and decreased invasiveness. In fact, the use of a SEMS protecting the skin tract could reduce the risk of wound-related complications, like infection and incisional hernia. Moreover, endoscopic necrosectomy is mainly performed under moderate conscious sedation and does not require general anesthesia. This is an important advantage also applicable to PEN, which leads to less systemic pro-inflammatory response and minimal collateral damages in already critically ill patients, thereby improving patients’ quality of life [12, 15, 25]. Although not yet addressed, PEN could be cost-effective and future studies should estimate its economic benefits.

Although both the TENSION and MISER trials showed no difference in terms of mortality between endoscopic and surgical step-up approach, patients undergoing endoscopic procedures had lower rate of disease-related adverse events (i.e., abdominal pain, infection), shorter length of ICU stay and hospitalization, and more economic advantages [4, 29]. Unfortunately, there is limited data about PEN through SEMS and its clinical outcomes, even if the available evidences are encouraging. In our experience, SEMS-assisted PEN was technically feasible, leading to the resolution of symptoms and stent removal in 20 days, in line with those previously reported [5, 7, 8, 22, 25]. Moreover, we did not experience any periprocedural or delayed adverse event, though we do not have to disregard the possible risks of a combined approach (i.e., bleeding, suprainfection, stent migration and stent occlusion due to the LAMS [30], abdominal pain).

Main concerns were about avoidance of a chronic pancreatic-cutaneous fistula (PCF) that could undermine long-term clinical outcomes and patient’s quality of life. As reported by Ross et al. [31], a dual-modality approach, in which endoscopic trans-enteric stents were placed into the necrotic collection immediately after percutaneous drainage, allowed redirection of pancreatic juice back into the gastrointestinal (GI) tract. This could decrease the risk of PCF development in patients with disconnected duct syndrome due to the severe pancreatic inflammation. In our case, the cutaneous fistula was conservatively treated and its closure was obtained after 2 months.

Only another case of WOPN drained using a dual endoscopic approach with LAMS and percutaneous SEMS has been reported [26]; in this report, however, the percutaneous drainage was made in two steps, with the esophageal SEMS placed 4 days after the percutaneous access achievement. In our study, instead, the percutaneous procedure was made in a single step, allowing to rapidly gain a large bore gateway to perform endoscopic necrosectomy in the same session. Moreover, the percutaneous stent turned out to be a convenient access to perform high-volume lavages using saline solution and intracystic instillation of antibiotics and antimicrobial agents.

In our opinion, this approach appears to be safe and perfectly suits the extended concept of “dual-approach,” overcoming the limits of both endoscopy and radiology and highlighting the need for multidisciplinarity in this particular setting of patients. However, there are several limitations, mainly due to the lack of high-quality evidence. In fact, the dual-endoscopic technique, especially for PEN, needs to be further refined, with determination of the optimum interval between sessions, end-point during each session, and the final end-point. Furthermore, it should be performed in referral centers, with availability of expert endoscopists, interventional radiologists, and surgical facilities.

## Conclusions

Patients affected by WOPN with deep extension of the necrosis are “hard-to-treat” patients, and a dual approach using LAMS and percutaneous, large bore SEMS is a compelling option of treatment that could maximize debridement volume and reduce the need for surgery. Further studies are needed to define the clinical outcome and the cost-effectiveness of this approach.

## Data Availability

All data generated or analyzed during this study are included in this published article.

## Abbreviations

EUS: Endoscopic ultrasound
WOPN: Walled-off pancreatic necrosis
PEN: Percutaneous endoscopic necrosectomy
SEMS: Self-expandable metal stent
MOF: Multiorgan failure
ANP: Acute necrotizing pancreatitis
LAMS: Lumen apposing metal stent
DEN: Direct endoscopic necrosectomy
CT: Computed tomography
PFCs: Pancreatic fluid collections
ICU: Intensive care unit
VARD: Video-assisted retroperitoneal debridement
RCTs: Randomized control trials
PCD: Percutaneous drainage
PCF: Pancreatic-cutaneous fistula
GI: Gastrointestinal
